# Alternative Splicing and Nonsense-Mediated RNA Decay Contribute to the Regulation of *SHOX* Expression

**DOI:** 10.1371/journal.pone.0018115

**Published:** 2011-03-23

**Authors:** Claudia Durand, Ralph Roeth, Harsh Dweep, Irena Vlatkovic, Eva Decker, Katja Ute Schneider, Gudrun Rappold

**Affiliations:** 1 Department of Human Molecular Genetics, University of Heidelberg, Heidelberg, Germany; 2 Medical Research Center, Faculty of Medicine Mannheim, University of Heidelberg, Mannheim, Germany; 3 Research Center for Molecular Medicine of the Austrian Academy of Sciences, Vienna, Austria; Southern Illinois University School of Medicine, United States of America

## Abstract

The human *SHOX* gene is composed of seven exons and encodes a paired-related homeodomain transcription factor. *SHOX* mutations or deletions have been associated with different short stature syndromes implying a role in growth and bone formation. During development, *SHOX* is expressed in a highly specific spatiotemporal expression pattern, the underlying regulatory mechanisms of which remain largely unknown. We have analysed *SHOX* expression in diverse embryonic, fetal and adult human tissues and detected expression in many tissues that were not known to express *SHOX* before, e.g. distinct brain regions. By using RT-PCR and comparing the results with RNA-Seq data, we have identified four novel exons (exon 2a, 7-1, 7-2 and 7-3) contributing to different *SHOX* isoforms, and also established an expression profile for the emerging new *SHOX* isoforms. Interestingly, we found the exon 7 variants to be exclusively expressed in fetal neural tissues, which could argue for a specific role of these variants during brain development. A bioinformatical analysis of the three novel 3′UTR exons yielded insights into the putative role of the different 3′UTRs as targets for miRNA binding. Functional analysis revealed that inclusion of exon 2a leads to nonsense-mediated RNA decay altering *SHOX* expression in a tissue and time specific manner. In conclusion, *SHOX* expression is regulated by different mechanisms and alternative splicing coupled with nonsense-mediated RNA decay constitutes a further component that can be used to fine tune the *SHOX* expression level.

## Introduction

The human *SHOX* gene resides in the pseudoautosomal region 1 on the short arm of the X and Y chromosome. Like all genes in the pseudoautosomal region, it escapes X inactivation and therefore shows a “pseudo-autosomal” inheritance pattern [1]. *SHOX* encodes for a paired-related homeodomain transcription factor. Homeodomain transcription factors are involved in the regulation of pattern formation, differentiation and organogenesis [2], and deficiencies in these genes can lead to a misregulation of developmental processes resulting in malformations [3], [4]. The heterozygous loss of *SHOX* function due to deletions or mutations has been shown to cause Leri-Weill Dyschondrosteosis (LWD) while homozygous loss leads to Langer mesomelic dysplasia [5], [6]. In addition, *SHOX* defects are a major cause of Idiopathic Short Stature (ISS) and are involved in the etiopathology of Turner Syndrome [7], [8]. In these syndromes, *SHOX* defects are implicated in inaccurate bone development and longitudinal body growth. Studies in human and chicken revealed specific *SHOX* expression in the pharyngeal arches and the early developing limbs during embryonic and fetal development, consistent with the symptoms seen in Turner and Langer syndrome as well as LWD [7], [9].

To date, there are seven known *SHOX* exons encoding for two different isoforms - *SHOXa* and *SHOXb* that employ different 3′ exons (exon 6a or 6b). *SHOXa* and *SHOXb* encode proteins of 292 and 225 amino acids, respectively [1]. Alternative usage of two different promoters (one residing in front of exon 1 and one residing at the beginning of exon 2) leads to two mRNAs differing in the 5′UTR but generating identical proteins [10]. The homeodomain, which is responsible for the DNA binding of this transcription factor, is encoded by exons 3 and 4. SHOXa encompasses an OAR domain (**o**tp, **a**ristaless, and **r**ax) with a transactivating function, while this domain is lacking in SHOXb [1].

*SHOX* expression is found to be tightly regulated by different mechanisms to obtain the *SHOX* specific spatio-temporal expression pattern, for example by usage of the two different promoters [10], by enhancer regions residing up- and downstream of the gene [11], [12], [13], [14] and by alternative usage of the two different 3′ exons [1]. In our study, we investigated if *SHOX* possibly features additional coding or regulatory capacities that have not been identified so far.

## Methods

### Origin of RNA and Reverse Transcription of RNA

Total RNA from various human adult tissues was purchased from Ambion, embryonic and fetal RNA was kindly provided by the MRC-Wellcome Trust Human Developmental Biology Resource (HDBR, Newcastle, UK). Fetal RNA originated from tissues from two different fetuses of fetal week 2, embryonic RNA originated from a Carnegie Stage 16 embryo.

RNA from cell lines was prepared using the illustra RNA spin Mini Kit (GE Healthcare) according to the manufacturer's protocol. The following cell lines and cultured primary cells were used: L87/4 (bone marrow fibroblasts [15]), NHDF and HDF (primary human fibroblasts, Promocell) and Hs27 (human fibroblasts, ATCC CRL-1634).

Reverse transcription was performed with Superscript III Reverse Transcriptase using random hexamer and oligodT primers (Invitrogen) with 1 µg of adult or cell line RNA. Reverse transcription of fetal/embryonic RNA was carried out with 200 ng RNA as a template.

### Tissue screening PCRs

Screening of cDNAs for different splice variants was performed with intron spanning exonic primers listed in Table 1. PCR amplicons were designed to specifically detect a single *SHOX* isoform at a time. PCR experiments were carried out in a 25 µl volume with 2 µl cDNA as a template in a PTC-200 Thermocycler (MJ Research). All primers were designed for an annealing temperature of 60°C.

**Table 1:** Primers used for the cDNA screening for *SHOX* splice variants.

Primer Name	Sequence (5′→3′)	Exon Location
SHOX Ex2 For	CCGGTGCATTTGTTCAAGGA	2
SHOX Ex4/5 Rev	TGCCCAAGATGACGCCTTTA	4/5
SHOX Ex2a For	CGGAGATCACGGGAAGACT	2a
SHOX cDNA For	ACGTCAACATGGGAGCCTTA	5
SHOX 3′UTR For	ACCGCTGTAAAATGACGGAG	6a
SHOX 3′UTR Rev	TACCCACGTGTGTCGAAGAA	7 variant 1
SHOX Ex7/2 Rev	TAGGAGAATGAGGGCGTCAC	7 variant 2
SHOX Ex7new Rev1	AAGTGGAAAAACGGGTGTTG	7 variant 3
ARF For	GCCAGTGTCCTTCCACCTGTC	1
ARF Rev	GCCTCGTTCACACGCTCTCTG	3

PCR experiments for the screening and detection of different exons of *SHOX* were carried out using HotStarTaq DNA polymerase (Qiagen) under the following conditions: initial denaturation at 95°C for 15 min followed by 40 cycles each consisting of 30 sec at 94°C, 30 sec at 60°C and 30–60 sec at 72°C followed by one cycle of 5 min at 72°C. For the housekeeping gene ADP-ribosylation factor 1 (ARF1), only 35 cycles were carried out.

Resulting PCR products were checked for specificity by directly sequencing them on a MegaBACE sequencer using the DYEnamic ET Terminator Cycle Sequencing Kit (GE Healthcare) according to the manufacturer's instructions.

### Rapid amplification of cDNA ends (RACE)

To determine the 3′ end of novel *SHOX* transcripts containing exon 7 variants, 3′RACE experiments were carried out using the GeneRacer™ Kit (Invitrogen) according to the manufacturer's instructions. For the RACE-PCR, we used the following primers: SHOXRaceEx7For 5′-TTGAAAGGGGATGTGGCTTCACGA-3′ and SHOXRaceEx7nestedFor 5′-TCTGTTATTGTCGGCAGGCGGTGAG-3′.

### Cell culture and inhibition of nonsense-mediated RNA decay

Primary normal human dermal fibroblasts (NHDF) were cultured in DMEM high glucose medium (Gibco/Invitrogen) supplemented with 10% fetal bovine serum (PAA) and 1% Penicillin/Streptomycin (Gibco/Invitrogen) at 37°C and 5% CO_2_. Cells were grown to 80–90% confluence and then treated with 100 µg/ml cycloheximide (Sigma) for 6 h or 20 mM Wortmannin (Sigma) for 2 h, respectively.

### Quantitative PCR

Quantitative real-time PCR (qRT-PCR) was carried out using the Applied Biosystems 7500 Real-Time PCR System and Absolute SYBR Green ROX Mix (Abgene). Amplification of the exon 6a/7-1 boundary was carried out with QuantiFast SYBR Green PCR Kit (Qiagen). Each sample was run in duplicates. Relative levels of mRNA expression were calculated according to the delta-delta Ct method [16] by normalization to the expression of two different housekeeping genes (succinate dehydrogenase complex subunit A (*SDHA*) and peptidylprolyl isomerase A (*PPIA*)). PCR amplifications were carried out with the following primers: SHOXafor 5′-CCTACGTCAACATGGGAGCCTTAC-3′, SHOXarev 5′-CCCGAAGGGCGGCGGG-3′, PPIAfor 5′-CGGGAGGCCAGGCTCGT-3′, PPIArev 5′-TGAAAGCAGGAACCCTTATAACCAA-3′, SDHAfor 5′-TGGGAACAAGAGGGCATCTG-3′, SDHArev 5′-CCACCACTGCATCAAATTCATG-3′. For the amplification of exon 2a or exon 7-1 specific products, primers listed in Table 1 were used.

## Results

### Identification of novel *SHOX* exons

**Figure 1:**
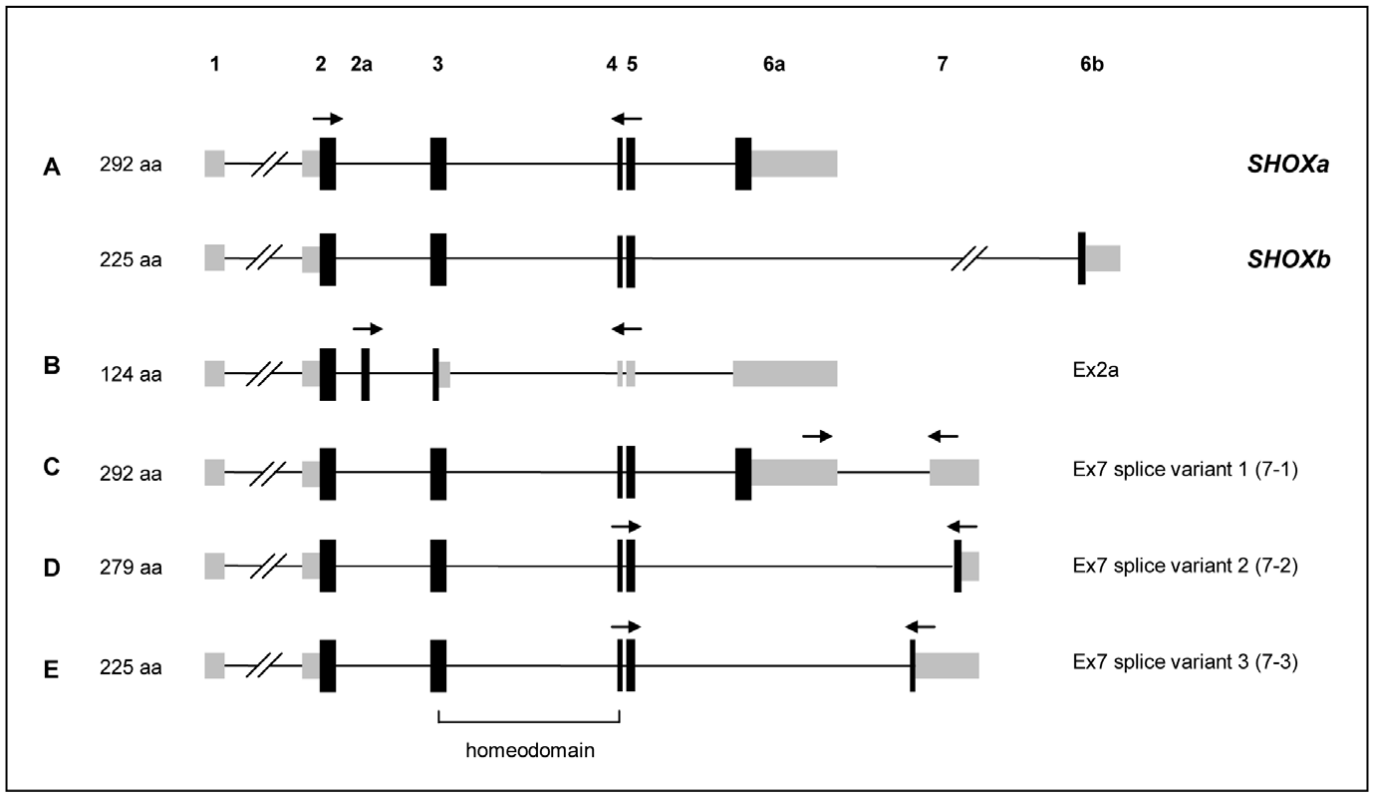
Schematic representation of known and novel *SHOX* splice variants. Grey depicts untranslated regions, black depicts open reading frame. (A) *SHOXa* and *SHOXb* as described in the literature [1]. (B) Insertion of exon 2a leads to a premature stop codon in exon 3. (C) Addition of novel exon 7 elongates the 3′UTR of the *SHOX* transcript. (D, E) Exon 7 (with alternative 5′ splice sites) can be attached directly to exon 5 and become part of the open reading frame. Arrows represent position of primers used for the detection of the respective splice variants in the tissue screening.

**Figure 2:**
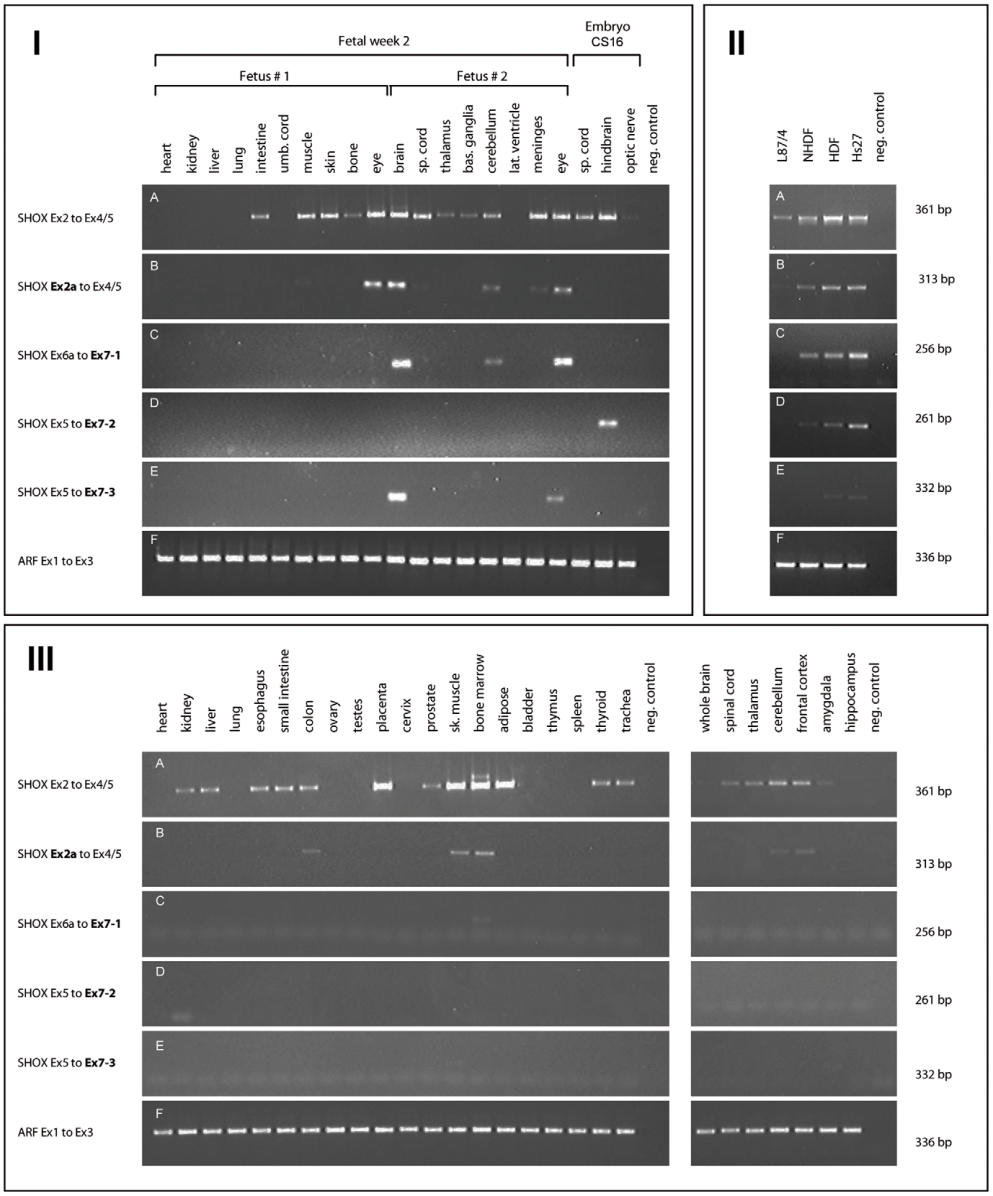
Screening for the different *SHOX* transcripts. **(I) Screening of fetal and embryonic tissues.** (A) Detection of different *SHOX* isoforms by using a forward primer situated within exon 2 and a reverse primer located at the exon junction of exons 4 and 5. Strongest expression is seen in muscle and skin and in different parts of the brain. (B) Detection of exon 2a containing transcripts using primers situated in exon 2a and exons 4/5. Expression of this splice form is restricted to eye and brain. (C) Detection of *SHOX* isoforms containing exon 7-1 using primers spanning exon 6a to exon 7-1. Expression is restricted to brain and eye. Expression in the eye varies between fetus # 1 and fetus # 2. (D) Expression of *SHOX* isoforms containing exon 7-2 is restricted to embryonic hindbrain. (E) Detection of *SHOX* transcripts containing exon 7-3. Expression is restricted to brain and eye and varies between fetus # 1 and fetus # 2. Exon 7-2 and exon 7-3 were detected by primer pairs spanning exon 5 to the 5′ends of the respective variants of exon 7. (F) Expression of the housekeeping gene *ARF1* was used to indicate similar mRNA levels. **(II) Screening of cell lines and cultured cells that show** ***SHOX*** **expression.** L87/4 bone marrow stromal cell line; NHDF normal human dermal fibroblast primary cells; HDF human dermal fibroblast primary cells; Hs27 diploid human fibroblasts. (A) All fibroblasts tested express *SHOX* at different levels. (B, C, D) Primary cultured fibroblasts express different *SHOX* isoforms containing exon 2a, exon 7-1 and exon 7-2, respectively. (E) The exon 7-3 containing isoform is absent in all cells tested. (F) Expression of the housekeeping gene *ARF1* was used to indicate similar mRNA levels. **(III) Screening of adult tissues.** (A) *SHOX* is expressed in a variety of tissues with strongest expression in placenta, skeletal muscle, bone marrow and adipose tissue. *SHOX* is also expressed in various brain tissues tested. (B) Detection of transcripts that include exon 2a. Expression is found in skeletal muscle and bone marrow and also weakly in the cerebellum and frontal cortex. (C–E) Detection of isoforms containing the different exon 7 variants; expression was not found for any of the tissues tested. (F) Expression of the housekeeping gene *ARF1* was used to indicate similar mRNA levels.

A systematic RT-PCR screen of 48 different human tissues (3 embryonic, 18 fetal and 27 adult) and four cell lines was first carried out to analyse the expression of the known *SHOX* isoforms *SHOXa* and *SHOXb* using a primer pair spanning from exon 2 to exon 4/5 (Figure 1A). In embryonic and fetal tissue, strongest expression was seen in muscle, skin and several neural tissues like brain, spinal cord, eye and meninges. We also showed expression in distinct subregions of the brain such as hindbrain (cerebellum), thalamus and basal ganglia (Figure 2 IA). In adult tissue, strongest expression was found in bone marrow, adipose tissue, placenta and skeletal muscle. Similar to the findings in fetal tissue, *SHOX* was also expressed in the brain (thalamus, cerebellum, frontal cortex) (Figure 2 IIIA). *SHOX* expression in specific human brain regions had not been reported before.

These RT-PCRs also revealed additional bands (e.g. in bone marrow) that differed from the expected band size of 361 bp (Figure 2 IIIA). Sequencing of this band indicated that 88 additional nucleotides were included into the *SHOX* cDNA between exon 2 and exon 3, which we termed exon 2a, according to the position in the cDNA (Figure 1B). Sequence and genomic position of exon 2a is given in Figure S1. Inclusion of exon 2a into the mRNA causes a frameshift and a premature stop codon in exon 3. Thus, a predicted resulting protein would be truncated, lack a homeodomain and consist of 124 amino acids (Figure 1B). Comparison of the *SHOX* genomic region in different species (UCSC browser http://genome.ucsc.edu/ and ECR browser http://ecrbrowser.dcode.org/) revealed that, unlike the formerly known *SHOX* exons, the novel 88 bp exon is not conserved between vertebrate species (data not shown).

This comparative analysis furthermore indicated an additional conserved genomic region downstream of exon 6a. We carried out PCR from cDNA of several tissues using a forward primer located in exon 6a and a reverse primer within the conserved region spanning a genomic distance of 4193 bp. The resulting PCR product consisted of only 256 bp and sequencing revealed that the conserved region, that we then termed exon 7 splice variant 1 (7-1),can be spliced directly to the 3′ end of exon 6a, resulting in an elongated 3′ UTR (Figure 1C). To confirm the 3′ end of this novel *SHOX* splice variant, we carried out 3′RACE experiments and located the end of exon 7 at position chrX/Y: 532,318 according to NCBI36/hg18 (data not shown).

Using primers spanning exon 5 to 7, we found another two novel alternative 5′ splice sites of exon 7 leading to two additional *SHOX* isoforms (Figure 1D, 1E). These two exon 7 variants (exon 7-2 and exon 7-3) are directly attached to exon 5 and thus become part of the open reading frame of *SHOX*, while exon 6 is lacking. To verify that exon 7-2 and 7-3 are indeed part of a complete *SHOX* mRNA, we carried out PCR using primers residing in exon 2 and exon 7 and were able to detect full *SHOX* transcripts comprising exon 2 to 5 and the exon 7 variants (data not shown).

We therefore conclude that the four identified novel exons result in at least four different *SHOX* isoforms, an overview of which is given in Figure 1. DNA sequences, genomic positions of the novel exons and a comparison of the amino acid sequences of the (hypothetically) encoded protein isoforms are annotated in Figure S1.

### Screening and functional analysis of the novel splice variants

To analyse where and when the novel *SHOX* splice variants are expressed, we carried out RT-PCR from cDNAs of the 48 human tissues described above. The exon 2a containing transcript was detected in several fetal and adult tissues with strongest expression in fetal eye and brain, adult bone marrow and skeletal muscle and in most of the cell lines tested (Figure 2 IB to 2 IIIB).

The three exon 7 splice variants were expressed in several embryonic and fetal tissues (Figure 2 I) as well as in cultured dermal fibroblasts (NHDF, HDF and Hs27) (Figure 2 II), but not in any of the adult tissues (Figure 2 III). Thus, these isoforms may serve a special function during early development.

#### Exon 7 – the impact of alternative splicing on miRNA binding

The 3′UTR of a gene represents the main target site for miRNAs that mediate posttranscriptional gene silencing by annealing to specific sequences within the 3′UTR of target mRNAs [17]. Therefore, the novel exon 7 variants might comprise additional or novel binding sequences for miRNAs that may regulate SHOX expression. To address this issue, we carried out a comprehensive bioinformatical analysis of the different 3′UTR sequences of the different *SHOX* isoforms using the miRWalk algorithm with miRBase release 14.0 [18].

The highest absolute number of binding candidates was found in exon 6a, which displays the longest of all known *SHOX* 3′UTRs (Table 2). Also the average density (average number of binding sites per 100 bp) of predicted binding sites is comparatively high for this exon and is only surpassed by exon 6b (Table 2). The binding site density of exon 7-1 and 7-2 is lower than the ones of exon 6a and 6b while exon 7-3 is comparable to exon 6a. Usage of exon 7-2 instead of exon 6a or 6b therefore leads to transcripts with a lower predicted miRNA binding site density. Binding sites with a seed length longer than 10 bp (arguing for a high specificity of prediction) also only have been predicted for exon 6a, 6b and 7-3, with exon 6a exhibiting the highest number of these high seed length predictions. This may indicate that exon 7-2 can be used to escape the downregulation by miRNAs. An overview of predicted binding sites for each 3′UTR exon is given in Table S1. Statistical analysis (one-way ANOVA) also indicates that the means of binding sites are significantly different among the five *SHOX* 3′UTR exons (p<0.001) (Table S2a). Thereafter, Student's t-test was performed to compare the means of all pairs with a statistical significance level (a = 0.05). The mean of binding sites of exon 6a was found to be significantly different in a pairwise comparison with exon 6b, exon 7-1, exon 7-2 and exon 7-3, i.e. p = 0.0134, p<0.0001, p = 0.0006 and p<0.0001, respectively, suggesting a distinct role of exon 6a in miRNA regulation (Table S2b).

**Table 2:** Overview of *SHOX* exon lengths and number of predicted miRNA binding sites.

Exon	Length of Exon (bp)	Total number of predicted binding sites	Binding site density
6a	2433	329	13.5
6b	627	108	17.2
7-1	823	62	7.5
7-2	337	29	8.6
7-3	1642	219	13.3

Binding site density is calculated as the average number of predicted miRNA binding sites per 100 bp.

#### Exon 2a and exon 7-1 – alternative splicing and nonsense-mediated RNA decay

Inclusion of exon 2a into the *SHOX* mRNA results in a frameshift leading to a premature termination codon (PTC) within exon 3, which renders exon 4, 5 and 6 as part of the 3′UTR of the transcript. Messenger RNAs containing a PTC are generally targeted for degradation by nonsense-mediated RNA decay (NMD) [19]. This mechanism prevents the translation of transcripts containing PTCs leading to truncated proteins or polypeptides that are potentially noxious for the cell or the organism. Termination codons are usually recognized as PTCs if they are located more than 50 nt upstream of the final exon-exon junction [20].

Besides the insertion of exon 2a, which leads to a premature stop codon in exon 3, also the addition of exon 7-1 to the *SHOXa* mRNA gives rise to an exon/exon junction more than 50 nt downstream of the original stop codon in exon 6a, suggesting that both isoforms could be a target of NMD.

To investigate if these two alternatively spliced isoforms are targeted by NMD, we pharmacologically blocked the NMD pathway and analysed whether the novel splice variants accumulated in the cells. We used two different NMD-blocking drugs: Wortmannin (WM), an inhibitor of the phosphatidylinositol 3-kinase-related protein kinase hSMG1, which is part of the NMD machinery [21], and Cycloheximide (CHX), an inhibitor of the translation process [22].

**Figure 3:**
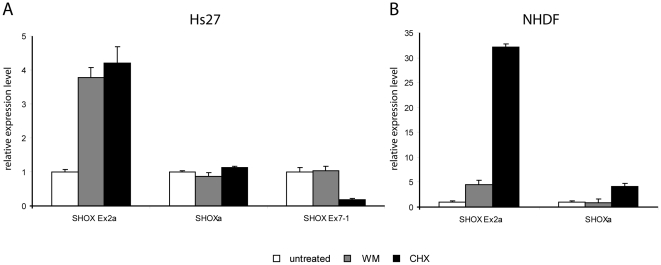
Effect of NMD inhibition on the expression levels of different *SHOX* isoforms. (A) Expression levels in Hs27 cells. Left: The relative amount of exon 2a containing mRNA in HS27 cells increases after NMD inhibition by CHX or WM. Middle: After addition of CHX or WM, *SHOX*a levels remain unchanged. Right: Addition of WM does not change the expression level of exon 7-1 containing mRNA; addition of CHX leads to a decrease of expression. (B) Expression levels in NHDF cells. Left: The relative amount of exon 2a containing mRNA in NHDF increases after NMD inhibition by CHX or WM. Right: After addition of WM, *SHOXa* levels remain unchanged; addition of CHX leads to a slight increase of *SHOX* (4.1x). However, in comparison to the increase seen for exon 2a (32.2x), this increase is negligible. Relative expression levels of untreated cells were always set to one.

To analyse the effect of blocking the NMD pathway, NHDF primary cells and Hs27 cells were treated with CHX or WM or left untreated. We then analyzed the expression levels of the exon 2a and exon 7-1 containing *SHOX* isoforms by qRT-PCR (Figure 3). The isoform containing exon 2a could be detected in both cell lines. Treatment with either WM or CHX led to an increase of exon 2a containing mRNA in NHDF and Hs27 cells, indicating that this isoform is normally degraded by NMD (Figure 3A and B, left graph). To confirm the specific effect of CHX and WM treatment, we also evaluated the expression level of the most prominent isoform, *SHOXa*, which does not contain a PTC (Figure 3 A, middle graph, and 3 B, right graph). In Hs27 cells, the *SHOXa* level remained unaltered upon WM or CHX treatment (Figure 3A, middle graph), indicating a very specific effect of CHX and WM on RNA containing *SHOX* exon 2a in this cell line. In NHDF cells, addition of CHX, but not of WM led to an increase of the *SHOXa* expression level (Figure 3 B, right graph) indicating a slightly unspecific reaction upon CHX treatment. However, the increase seen for *SHOX* Exon 2a was to a considerable degree higher (32.2x vs. 4.1x). Thus, we assume that, even when taking into consideration some unspecific CHX effect, CHX leads to a specific inhibition of NMD of the exon 2a containing *SHOX* isoform. Our data derived from Hs27 and NHDF cells therefore argue for a depletion of the exon 2a containing isoform by NMD.

As the expression level of the exon 7-1 containing isoform was not sufficient for reliable detection by qRT-PCR in NHDF, we analyzed this isoform only in Hs27 cells. NMD inhibition by WM or CHX failed to increase the level of exon 7-1 containing mRNA (Figure 3 A, right graph), suggesting that despite the presence of a PTC this isoform is not subjected to NMD.

## Discussion

### *SHOX* expression pattern and identification of novel *SHOX* exons

We have analysed the expression pattern of the *SHOX* gene by RT-PCR in a variety of embryonic, fetal and adult tissues and cell lines. Besides the known *SHOX* expression in tissues involved in body growth such as chondrocytes and cartilage, *SHOX* is expressed in various other additional tissues, e.g. in different fetal and adult brain regions such as hindbrain (cerebellum), thalamus and basal ganglia, pointing to an additional function of *SHOX* during fetal brain development and maintenance of brain functions. However, obvious brain malformations or cognitive developmental delay have not been described in patients with LWD, Turner or Langer syndrome or ISS patients with *SHOX* haploinsufficiency. We therefore speculate that *SHOX2*, a highly related *SHOX* paralogue [23] that is also expressed in the brain, may partly take over the functions of *SHOX* in the developing brain in these patients. Support for this hypothesis comes from the expression patterns of these two genes in chicken brain where, like in human, both genes are expressed (there is no *SHOX* orthologue in rodents). Whereas *SHOX/Shox* and *SHOX2/Shox2* expression patterns only partially overlap in the developing limb [7] [9], the area of *Shox* expression is completely covered by the broader *Shox2* expression pattern in the developing chicken brain (Figure S2).

We have identified four novel *SHOX* exons, which create new coding and untranslated regions. Similar to the known isoforms *SHOXa* and *SHOXb* with important functions during limb development, most of the novel splice variants are only expressed very weakly in most tissues. This low expression abundance is a feature of many transcription factors that need to recognize the correct target sites within the genome and to react to regulatory events [24].

Exons 2a and 7 were identified by RT-PCR and validated by subsequent sequencing. To also confirm these data by RNA-Sequencing (RNA-Seq), we searched published RNA-Seq data [25], [26], [27], and data integrated into the UCSC browser NCBI36/hg18 (Burge RNA-Seq [28], CSHL Long RNA-Seq, GIS RNA-Seq, Caltech RNA-Seq and Helicos RNA-Seq) that had been performed on 11 tissues and 15 cell lines including those known by RT-PCR to express novel *SHOX* exons. These data showed very low densities of mapped reads in the whole *SHOX* genomic region probably due to the low expression level of *SHOX* in tested cells and tissues and did not provide conclusive information about known or novel *SHOX* isoforms. As *SHOX* expression is comparatively higher in human fibroblasts (Figure 2), we further analysed an RNA-Seq dataset of Hs27 human fibroblast cells (Irena Vlatkovic and Denise Barlow, unpublished data; manuscript in preparation). This Hs27 RNA-Seq showed expression of known *SHOX* exons and exons 2a and 7 (data not shown) further validating the existence of the novel exons.

The exon 2a containing isoform is expressed in both fetal and adult tissues whereas all exon 7 containing isoforms are present in fibroblasts but otherwise restricted to embryonic and fetal stages, pointing to a likely role of exon 7 during early development.

### Alternative splicing as a possibility to regulate gene expression

Alternative splicing does not only increase the proteome of an organism [28], but also contributes to the complex regulation of the levels and tissue specificity of gene expression (for a review on the different splicing mechanisms see [29]). Based on our results, we suggest that the alternative splicing of the *SHOX* gene principally exerts regulatory functions. First, *SHOX* possesses two different promoters that generate transcripts with identical coding capacity but differing in the 5′UTR leading to different translational efficiencies of the transcript [10]. Second, *SHOX* is known to be expressed as two alternative isoforms, *SHOXa* and *SHOXb* (Figure 1A), with the latter lacking the OAR domain with a supposed transactivating function. The SHOXb protein might therefore act as a regulatory modulator of SHOXa activity [30]. Third, we have now identified additional *SHOX* isoforms, which are all likely to be involved in the fine tuning of the regulation of *SHOX* expression. The novel *SHOX* isoforms containing exon 7-2 or 7-3 do not encode for an OAR domain and thus might also exert a regulatory role by modulating SHOXa activity.

The three exon 7 isoforms could also exhibit novel binding sites for miRNAs. As the majority of validated mRNA-miRNA interactions usually takes place in the 3′UTR of a gene [31], we have analysed the 3′UTRs of the different *SHOX* splice variants for miRNA binding sites by the miRWalk prediction tool. The absolute number of predicted binding sites is highest in exon 6a, which gives rise to the longest *SHOX* 3′UTR known to date, while the binding site density is highest in exon 6b. Long 3′UTRs and high binding site densities are characteristic for genes whose expression is regulated by miRNAs [32]. Exon 6a also contains the highest number of sequence motifs where binding was predicted to occur with a seed length longer than 10 bp, and statistical analyses showed that the means of binding sites in this exon is significantly different from the other *SHOX* 3′UTR exons. miRNA regulation of *SHOX* might therefore open up additional possibilities for the fine tuning of *SHOX* expression, and we assume that this regulation could mainly be accomplished via exon 6a and less likely by the exon 7 variants. Interestingly, we found exon 7 variants to be exclusively expressed in fetal neural tissues (Figure 2), suggesting that these variants might be of special importance during early brain development.

Inclusion of exon 2a into the *SHOX* mRNA leads to a transcript with a premature stop codon that most likely does not encode for a functional protein but undergoes degradation by nonsense-mediated RNA decay (Figure 3). Premature stop codons lead to NMD and can be generated by point or frameshift mutations, but also by alternative splicing, e.g. by inclusion/skipping of exons (as it is seen for *SHOX* exon 2a), by usage of alternative splice sites or by addition of a 3′UTR exon that leads to an exon/exon junction more than 50 nt downstream of the original stop codon (as seen for *SHOX* exon 7-1), which will then be recognized as premature [33].

Whereas we provide evidence for a degradation of the exon 2a containing *SHOX* isoform, the isoform containing exon 7-1 most likely escapes the degradation by NMD despite the presence of a PTC according to the 50 nt rule. NMD-resistant PTC-containing mRNAs have been previously reported for several genes, including β-globin and the familial Mediterranean fever gene [34], [35]. The definite mechanism that confers NMD-resistance still remains unanswered although it has been suggested that spatial rearrangements of the 3′UTR that affect the distance between PTC and poly(A) tail can modulate the NMD pathway [36]. The coupling of alternative splicing and NMD is a previously described phenomenon [e.g. 35,37], which is now also demonstrated for the *SHOX* gene for the exon 2a containing isoform. However, it is controversial whether the occurrence of the alternatively spliced products reflects constitutive unproductive splicing, hence cellular noise, or if this process provides an additional level of regulation to help the cell to achieve the proper level of expression for a given protein [33], [38]. If the first hypothesis is true, it would mean that the ratio of mRNAs encoding the functional or the non-functional protein is not significantly variable but that a certain amount of pre-mRNA is always spliced unproductively. A comparison of the expression of total *SHOX* mRNA with the expression of exon 2a containing mRNA (Figure 2 I-III A, B) clearly shows that this is not the case for *SHOX*. In fetal eye and brain, relatively high amounts of *SHOX* are detectable and there is also a detectable amount of the alternative exon 2a containing transcript, while this is not the case for e.g. fetal muscle or spinal cord, thus arguing for tissue-specific alternative splicing events. This time and tissue dependent specific splicing might be caused by the presence of different regulatory splicing factors that act in a manner autologous to transcription factors to down-regulate the expression of a gene by alternatively splicing the pre-mRNA to a product that undergoes NMD (also described in [39]).

Taken together, our results add important novel aspects to the complex regulation of the *SHOX* gene. In addition to two alternative promoters [10] and enhancers up- and downstream of the gene [11], [12], [13], [14], we have revealed that alternative splicing coupled with NMD can also contribute to the time and tissue specific regulation of *SHOX* expression and that usage of different 3′UTRs might also be involved in *SHOX* regulation. Only the orchestrated interaction of the different regulatory components ensures the accurate expression of *SHOX* during limb and brain development and in mature organisms.

## Supporting Information

Figure S1 **Novel** ***SHOX*** **exons and isoforms – important features.** (A) **DNA sequence and genomic location of the novel** ***SHOX*** **exons**. Capital letters indicate exonic sequence, small letters indicate flanking intronic sequence. For exon 7-2 and 7-3, letters in bold print indicate coding sequences, normal letters indicate 3′UTR. Genomic position according to NCBI36/hg18. (B) **Exon-wise comparison of the protein sequences of the different SHOX isoforms**. The protein sequence of the exon 7-1 containing SHOX isoform is identical to SHOXa and therefore not included into the comparison.
(TIF)

Figure S2 **Adjacent transverse sections of a d6 chicken head as exemplary illustration of** ***Shox*** **and** ***Shox2*** **expression in the developing chicken brain.** *Shox2* is strongly expressed in the dorsal root ganglia and in the diencephalon, whereas *Shox* expression is only seen in the diencephalon and completely covered by *Shox2* expression. *drg*, dorsal root ganglia; *de*, diencephalon; *le*, left eye; *re*, right eye. Arrows indicate specific *Shox*/*Shox2* expression. chShox riboprobes were generated and digoxigenin labelled by in vitro transcription of a PCR product amplified using the following primers out of chicken cDNA: chiSHOX_1_For gagcttgggaactccgatt and chiSHOX_2_Rev ttcagacagtcccagcctct. *In situ* hybridizations on tissue sections were carried out as described in Decker et al. 2011. Reference Figure S2 Decker E, Durand C, Bender S, Roedelsperger C, Glaser A, Hecht J, Schneider KU, Rappold G (2011). *FGFR3* is a target of the homeobox transcription factor SHOX in limb development. Hum Mol Genet. doi:10.1093/hmg/ddr030.
(TIF)

Table S1 **Overview of predicted miRNA binding sites within the different 3′ exons of** ***SHOX*** **.** For each exon, name, position and seed length of each miRNA predicted to bind are given.
(XLS)

Table S2 **Statistical analyses of miRNA binding site predictions.** (A) Overview of statistical analysis of one-way ANOVA on *SHOX* exons. (B) Overview of comparisons of means for each pair using Student's t-test (t = 1.96423 and a = 0.05).
(XLS)

## References

[1] Rao E, Weiss B, Fukami M, Rump A, Niesler B (1997). Pseudoautosomal deletions encompassing a novel homeobox gene cause growth failure in idiopathic short stature and Turner syndrome.. Nat Genet.

[2] McGinnis W, Krumlauf R (1992). Homeobox genes and axial patterning.. Cell.

[3] Boncinelli E (1997). Homeobox genes and disease.. Curr Opin Genet Dev.

[4] Goodman FR (2002). Limb malformations and the human HOX genes.. Am J Med Genet.

[5] Belin V, Cusin V, Viot G, Girlich D, Toutain A (1998). SHOX mutations in dyschondrosteosis (Leri-Weill syndrome).. Nat Genet.

[6] Shears DJ, Vassal HJ, Goodman FR, Palmer RW, Reardon W (1998). Mutation and deletion of the pseudoautosomal gene SHOX cause Leri-Weill dyschondrosteosis.. Nat Genet.

[7] Clement-Jones M, Schiller S, Rao E, Blaschke RJ, Zuniga A (2000). The short stature homeobox gene SHOX is involved in skeletal abnormalities in Turner syndrome.. Hum Mol Genet.

[8] Rappold G, Blum WF, Shavrikova EP, Crowe BJ, Roeth R (2007). Genotypes and phenotypes in children with short stature: clinical indicators of SHOX haploinsufficiency.. J Med Genet.

[9] Tiecke E, Bangs F, Blaschke R, Farrell ER, Rappold G (2006). Expression of the short stature homeobox gene Shox is restricted by proximal and distal signals in chick limb buds and affects the length of skeletal elements.. Dev Biol.

[10] Blaschke RJ, Topfer C, Marchini A, Steinbeisser H, Janssen JW (2003). Transcriptional and translational regulation of the Leri-Weill and Turner syndrome homeobox gene SHOX.. J Biol Chem.

[11] Benito-Sanz S, Thomas NS, Huber C, Gorbenko del Blanco D, Aza-Carmona M (2005). A novel class of Pseudoautosomal region 1 deletions downstream of SHOX is associated with Leri-Weill dyschondrosteosis.. Am J Hum Genet.

[12] Durand C, Bangs F, Signolet J, Decker E, Tickle C (2010). Enhancer elements upstream of the SHOX gene are active in the developing limb.. Eur J Hum Genet.

[13] Sabherwal N, Bangs F, Roth R, Weiss B, Jantz K (2007). Long-range conserved non-coding SHOX sequences regulate expression in developing chicken limb and are associated with short stature phenotypes in human patients.. Hum Mol Genet.

[14] Fukami M, Kato F, Tajima T, Yokoya S, Ogata T (2006). Transactivation function of an approximately 800-bp evolutionarily conserved sequence at the SHOX 3′ region: implication for the downstream enhancer.. Am J Hum Genet.

[15] Thalmeier K, Meissner P, Reisbach G, Falk M, Brechtel A (1994). Establishment of two permanent human bone marrow stromal cell lines with long-term post irradiation feeder capacity.. Blood.

[16] Pfaffl MW (2001). A new mathematical model for relative quantification in real-time RT-PCR.. Nucleic Acids Res.

[17] He L, Hannon GJ (2004). MicroRNAs: small RNAs with a big role in gene regulation.. Nat Rev Genet.

[18] Griffiths-Jones S, Saini HK, van Dongen S, Enright AJ (2008). miRBase: tools for microRNA genomics.. Nucleic Acids Res.

[19] Nicholson P, Yepiskoposyan H, Metze S, Zamudio Orozco R, Kleinschmidt N (2010). Nonsense-mediated mRNA decay in human cells: mechanistic insights, functions beyond quality control and the double-life of NMD factors.. Cell Mol Life Sci.

[20] Nagy E, Maquat LE (1998). A rule for termination-codon position within intron-containing genes: when nonsense affects RNA abundance.. Trends Biochem Sci.

[21] Yamashita A, Ohnishi T, Kashima I, Taya Y, Ohno S (2001). Human SMG-1, a novel phosphatidylinositol 3-kinase-related protein kinase, associates with components of the mRNA surveillance complex and is involved in the regulation of nonsense-mediated mRNA decay.. Genes Dev.

[22] Gonzalez CI, Bhattacharya A, Wang W, Peltz SW (2001). Nonsense-mediated mRNA decay in Saccharomyces cerevisiae.. Gene.

[23] Blaschke RJ, Monaghan AP, Schiller S, Schechinger B, Rao E (1998). SHOT, a SHOX-related homeobox gene, is implicated in craniofacial, brain, heart, and limb development.. Proc Natl Acad Sci U S A.

[24] Vaquerizas JM, Kummerfeld SK, Teichmann SA, Luscombe NM (2009). A census of human transcription factors: function, expression and evolution.. Nat Rev Genet.

[25] Core LJ, Waterfall JJ, Lis JT (2008). Nascent RNA sequencing reveals widespread pausing and divergent initiation at human promoters.. Science.

[26] Pan Q, Shai O, Lee LJ, Frey BJ, Blencowe BJ (2008). Deep surveying of alternative splicing complexity in the human transcriptome by high-throughput sequencing.. Nat Genet.

[27] Sultan M, Schulz MH, Richard H, Magen A, Klingenhoff A (2008). A global view of gene activity and alternative splicing by deep sequencing of the human transcriptome.. Science.

[28] Wang ET, Sandberg R, Luo S, Khrebtukova I, Zhang L (2008). Alternative isoform regulation in human tissue transcriptomes.. Nature.

[29] Keren H, Lev-Maor G, Ast G (2010). Alternative splicing and evolution: diversification, exon definition and function.. Nat Rev Genet.

[30] Rao E, Blaschke RJ, Marchini A, Niesler B, Burnett M (2001). The Leri-Weill and Turner syndrome homeobox gene SHOX encodes a cell-type specific transcriptional activator.. Hum Mol Genet.

[31] Bartel DP (2004). MicroRNAs: genomics, biogenesis, mechanism, and function.. Cell.

[32] Stark A, Brennecke J, Bushati N, Russell RB, Cohen SM (2005). Animal MicroRNAs confer robustness to gene expression and have a significant impact on 3′UTR evolution.. Cell.

[33] Lareau LF, Brooks AN, Soergel DA, Meng Q, Brenner SE (2007). The coupling of alternative splicing and nonsense-mediated mRNA decay.. Adv Exp Med Biol.

[34] Danckwardt S, Neu-Yilik G, Thermann R, Frede U, Hentze MW (2002). Abnormally spliced beta-globin mRNAs: a single point mutation generates transcripts sensitive and insensitive to nonsense-mediated mRNA decay.. Blood.

[35] Grandemange S, Soler S, Touitou I (2009). Expression of the familial Mediterranean fever gene is regulated by nonsense-mediated decay.. Hum Mol Genet.

[36] Eberle AB, Stalder L, Mathys H, Orozco RZ, Muhlemann O (2008). Posttranscriptional gene regulation by spatial rearrangement of the 3′ untranslated region.. PLoS Biol.

[37] Cuccurese M, Russo G, Russo A, Pietropaolo C (2005). Alternative splicing and nonsense-mediated mRNA decay regulate mammalian ribosomal gene expression.. Nucleic Acids Res.

[38] Skandalis A, Uribe E (2004). A survey of splice variants of the human hypoxanthine phosphoribosyl transferase and DNA polymerase beta genes: products of alternative or aberrant splicing?. Nucleic Acids Res.

[39] Zhang X, Azhar G, Huang C, Cui C, Zhong Y (2007). Alternative splicing and nonsense-mediated mRNA decay regulate gene expression of serum response factor.. Gene.

