# Supplementary figures and images for: Alternative Splicing and Nonsense-Mediated RNA Decay Contribute to the Regulation of SHOX Expression
Source: PLoS One. 2011 Mar 23;6(3):e18115. doi: 10.1371/journal.pone.0018115 (PMC3063249; doi:10.1371/journal.pone.0018115)

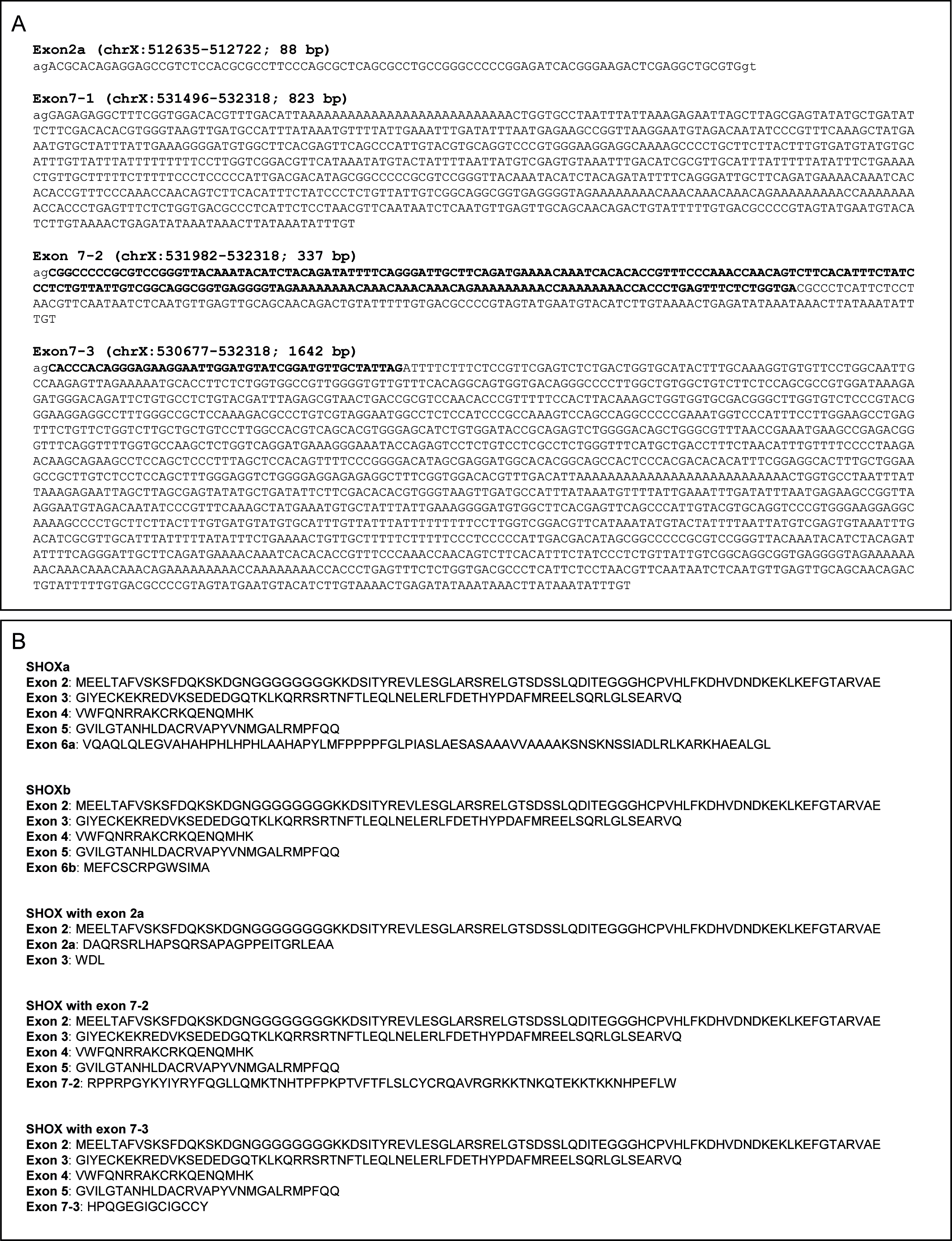

Supplement: Figure S1 — Novel SHOX exons and isoforms – important features. (A) DNA sequence and genomic location of the novel SHOX exons. Capital letters indicate exonic sequence, small letters indicate flanking intronic sequence. For exon 7-2 and 7-3, letters in bold print indicate coding sequences, normal letters indicate 3′UTR. Genomic position according to NCBI36/hg18. (B) Exon-wise comparison of the protein sequences of the different SHOX isoforms. The protein sequence of the exon 7-1 containing SHOX isoform is identical to SHOXa and therefore not included into the comparison. (TIF) [file pone.0018115.s001.tif]

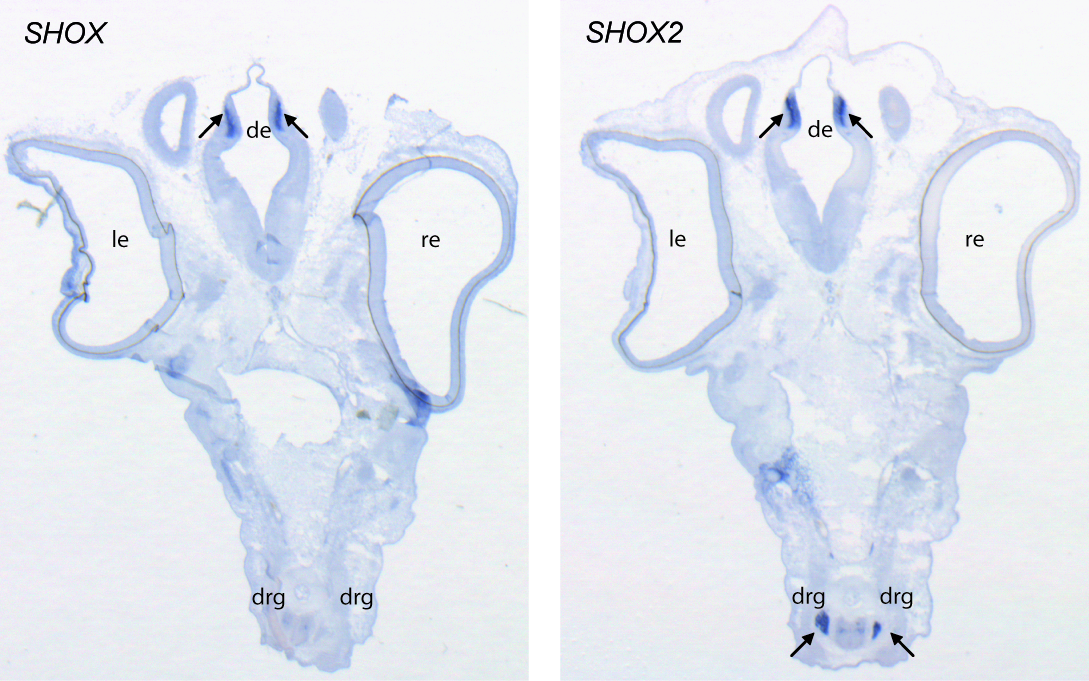

Supplement: Figure S2 — Adjacent transverse sections of a d6 chicken head as exemplary illustration of Shox and Shox2 expression in the developing chicken brain. Shox2 is strongly expressed in the dorsal root ganglia and in the diencephalon, whereas Shox expression is only seen in the diencephalon and completely covered by Shox2 expression. drg, dorsal root ganglia; de, diencephalon; le, left eye; re, right eye. Arrows indicate specific Shox/Shox2 expression. chShox riboprobes were generated and digoxigenin labelled by in vitro transcription of a PCR product amplified using the following primers out of chicken cDNA: chiSHOX_1_For gagcttgggaactccgatt and chiSHOX_2_Rev ttcagacagtcccagcctct. In situ hybridizations on tissue sections were carried out as described in Decker et al. 2011. Reference Figure S2 Decker E, Durand C, Bender S, Roedelsperger C, Glaser A, Hecht J, Schneider KU, Rappold G (2011). FGFR3 is a target of the homeobox transcription factor SHOX in limb development. Hum Mol Genet. doi:10.1093/hmg/ddr030. (TIF) [file pone.0018115.s002.tif]
